# Ultra-Processed Food Consumption and the Risk of Psoriasis: A Large Prospective Cohort Study

**DOI:** 10.3390/nu17091473

**Published:** 2025-04-27

**Authors:** Xinxing Peng, Xiangzi Li, Jiayu He, Min He, Ning Ning, Li Chen, Ping Yao, Yuhan Tang, Yanyan Li

**Affiliations:** 1 Shenzhen Center for Chronic Disease Control, Shenzhen Institute of Dermatology, No. 2021, Buxin Road, Luohu District, Shenzhen 518020, China; pongxinxing@163.com (X.P.); dr_lixz@163.com (X.L.); hejy666@126.com (J.H.); hemin0206@163.com (M.H.); 20nning@stu.edu.cn (N.N.); cl707339221@163.com (L.C.); 2 Department of Nutrition and Food Hygiene, Hubei Key Laboratory of Food Nutrition and Safety, Ministry of Education Key Laboratory of Environment and Health, School of Public Health, Tongji Medical College, Huazhong University of Science and Technology, Wuhan 430030, China; yaoping@mails.tjmu.edu.cn

**Keywords:** psoriasis, ultra-processed food, inflammation, obesity, diet

## Abstract

**Background:** The sales of ultra-processed food (UPF) are rapidly increasing worldwide, and there have been reports linking UPF consumption to several chronic diseases. However, there is limited prospective evidence exploring the impact of UPF on inflammatory skin diseases. **Objectives:** This study investigates the association between UPF intake and the incidence of psoriasis using data from the UK Biobank. **Methods:** UPFs were assessed based on the NOVA classification in this large prospective study. Cox proportional hazards regression was employed to estimate the association between UPF intake and the incident risk of psoriasis. Inflammation score (INFLA-score) and body mass index (BMI) were chosen as mediators to examine the mediation effect. Substitution analysis was performed to estimate the psoriasis risk when replacing the absolute amount of UPF with an equivalent weight of unprocessed or minimally processed food. **Results:** This study enrolled 121,019 participants aged 40–69 years from the UK Biobank. Over a 12-year (median) follow-up period, 1043 participants developed psoriasis. In the fully adjusted model, hazard ratios (95% confidence interval) for psoriasis across increasing quartiles of UPF consumption were 1.00 (reference), 1.07 (0.89, 1.28), 1.19 (1.00, 1.42), and 1.23 (1.03, 1.47), respectively (*p* for trend = 0.016). Factors such as age, sex, BMI, smoking status, drinking status, physical activity level, and Townsend Deprivation Index (TDI) did not significantly modify this association (*p* interaction > 0.05). The INFLA-score and BMI explained 6.5% (*p* = 0.012) and 30.5% (*p* < 0.001) of the association between UPF consumption and psoriasis risk, respectively. Replacing 20% of UPF weight in total diet with an equivalent proportion of unprocessed or minimally processed foods was associated with an 18% reduction in psoriasis risk (HR: 0.82; 95% CI: 0.72–0.94; *p* = 0.004). **Conclusions:** Our findings indicate that increased UPF consumption is associated with a higher risk of psoriasis. This provides valuable dietary guidance for preventing psoriasis and its related comorbidities.

## 1. Introduction

Psoriasis is a chronic inflammatory skin disease characterized by persistent plaque formation and epidermal exfoliation. Skin lesions can involve the entire body, particularly affecting areas such as palms, soles, nails, and even the genital region, often accompanied by varying degrees of pruritus or pain [1,2], which would seriously affect the quality of life of the patients. According to the most recent iteration of global disease burden estimation, the burden of psoriasis has remained relatively stable from 1990 to 2019. It affects approximately 2–3% of the global population, with incidence rates ranging from 30.3 per 100,000 person-years to 321.0 per 100,000 person-years among different areas [3,4]. In clinical practice, diverse treatments such as phototherapy, prescription drugs, and biologics are employed to manage psoriasis symptoms. Nevertheless, none of these treatments offers a cure for psoriasis, and some are accompanied by serious side effects. Moreover, psoriasis is linked to several comorbidities, including adverse cardiovascular outcomes (myocardial infarction, stroke, and cardiovascular mortality) [5], Crohn’s disease [6], and some psychological symptoms (depression, anxiety, and suicidal ideation) [7,8,9]. Considering both the physical and mental impact on patients, as well as the economic burden imposed by the disease on society, preventing psoriasis becomes crucial.

The pathogenesis of psoriasis remains incompletely understood. Previous research has revealed elevated serum levels of pro-inflammatory cytokines in psoriasis patients, potentially contributing to chronic systemic inflammation. Diet plays a crucial role in immunological modulation and inflammatory responses [10]. Alongside this, the rising consumption of ultra-processed foods may contribute to a pro-inflammatory state through various mechanisms [11]. A cross-sectional study based on the NHANES database found that UPF consumption was associated with IgE, childhood asthma, and eczema-related allergic symptoms [12]. The NOVA classification categorizes foods based on the degree of industrial processing they undergo, resulting in four groups: (1) unprocessed foods or minimally processed foods; (2) processed culinary ingredients; (3) processed foods; and (4) ultra-processed foods [13]. UPFs are characterized by changes in food matrix and texture, low nutritional quality, and the presence of contaminants resulting from processing, food additives, and other industrial ingredients. Statistical data indicate that ultra-processed foods contribute to over 50% of total energy intake in countries such as the US, Canada, and the UK [14]. Between 2006 and 2019, per capita UPF sales increased 6 kg in Australasia and North America and 9 kg in Western Europe, reflecting a global upward trend that continues to accelerate [15]. It is reported that a variety of noncommunicable diseases concomitant with psoriasis, for instance, cardiovascular disease (CVD) [16], type 2 diabetes (T2D) [17], and Crohn’s disease [18], are linked to the consumption of UPF. However, there is limited evidence regarding the role of UPF in psoriasis.

To prevent various diseases, diet stands out as one of the most controllable aspects of health management. Despite the high demand for dietary advice in psoriasis management, limited research has provided evidence regarding the role of diet [19]. Our study aims to investigate the relationship between the consumption of UPF and the risk of psoriasis, identify modifiable risk factors, and explore the underlying mechanisms.

## 2. Materials and Methods

### 2.1. Study Population

The UK Biobank is a large-scale population-based prospective cohort study that aims to explore the relative contributions of genetic susceptibility and exposures (including nutrition, lifestyle, medications, and so on) to disease development. The project began in 2006 and recruited 500,000 participants aged 40 to 69 years from across the UK, with roughly equal numbers of men and women. Basic participant information was collected through a touchscreen questionnaire and face-to-face interviews at 22 assessment centers during the baseline phase. In this study, a total of 125,080 participants who completed at least two 24 h dietary assessments were included. Participants with a history of psoriasis and those who withdrew from the follow-up were excluded. In consideration of the validity of the questionnaire, individuals with implausible energy intake (such as males with <800 kcal/day or >4200 kcal/day and females with <600 kcal/day or >3500 kcal/day) were also excluded. The final analytical sample comprised 121,019 participants.

### 2.2. Assessment of UPF Consumption

In this large prospective study, the validated web-based Oxford WebQ questionnaire was employed to collect dietary information. It presented 21 broad food groups and 200 commonly consumed foods and drinks [20], estimated the true longer-term diet considering different seasonal intakes and cohort ages, and performed well when compared with other tools assessed using the same statistical methodology [20].

Ultra-processed foods refer to those that have undergone complex industrial processes. They usually contain various synthetic additives, seasonings, and other non-natural ingredients and often possess a high level of convenience and a long shelf life. Such foods include soft drinks, mass-produced industrial-processed breads, sweet or savory packaged snacks, breakfast ‘cereals’, reconstituted meat products, and ready-to-eat/heat foods [21] (Table S1). In this study, we primarily focused on calculating the consumption of UPFs using a 24 h dietary questionnaire. Considering that UPFs typically exhibit characteristics such as high energy density and contain energy-free and weight-free food additives (such as emulsifiers and preservatives), we determined the proportion of grams of food intake from UPFs per day. This calculation was based on information regarding portion size for each food item and the number of servings consumed by the individual. Additionally, we further categorized individuals into UPF consumption quartiles.

### 2.3. Ascertainment of Psoriasis

The primary diagnostic data for psoriasis is collected from hospital admission records, following the International Classification of Diseases (ICD) codes. Specifically, the relevant ICD-10 codes include L400, L401, L403, L404, L405, L408, and L409, while the ICD-9 codes are 6960 and 6961. Additionally, other sources of psoriasis diagnostic information encompass self-reports, primary care records, and death registration data obtained through a survey (Field ID: 20002, 131742, and 131743). Censoring occurs at the time of death, withdrawal from the study, or the end of follow-up, whichever comes first.

### 2.4. Measurement of Covariates

Taking into account the potential confounding factors related to demographics and socioeconomic aspects, this study incorporated several covariates collected at baseline, including age, sex, ethnicity (white and others), BMI, total energy, TDI, physical activity, smoking status (never, former, current), drinking status (never, former, current), and INFLA-score. The estimation of total energy was based on food and beverage consumption from the previous day, excluding any nutritional supplements. Physical activity was quantified using MET minutes per week, calculated as follows: Total physical activity MET-minutes/week = 3.3 × walking minutes × walking days + 4.0 × moderate-intensity activity minutes × moderate days + 8.0 × vigorous-intensity activity minutes × vigorous-intensity days. The Townsend Deprivation Index was determined based on four factors: ‘households without a car’, ‘overcrowded households’, ‘households not owner-occupied’, and ‘persons unemployed’.

### 2.5. Statistical Analysis

The baseline characteristics of the study population were compared according to quartiles of UPF consumption. We conducted an analysis of variance test for continuous variables and a χ^2^ test for categorical variables. Next, we employed Cox proportional hazards regression models to estimate hazard ratios (HRs) and 95% confidence intervals (CIs) for the association between the weight proportion (%) of UPF in the total weight of food intake and the subsequent risk of psoriasis. The reference group was the first quartile of UPF consumption. Model 1 adjusted for demographic characteristics, Model 2 additionally included lifestyle factors, and the fully adjusted model (Model 3) further accounted for potential body weight-related confounders. Except for physical activity, which had a missing rate of 14.2%, all other covariates had a missing rate of less than 0.2%. When covariate information was lacking, we imputed median values for missing continuous variables. Additionally, we created a new category called ‘unknown’ for missing categorical variables.

We employed a restricted cubic spline with three knots at the 10th, 50th, and 90th percentiles to illustrate the dose–response relationship between UPF consumption and the hazard ratio of psoriasis risk. In this study, we hypothesize that psoriasis is indirectly mediated through chronic low-grade inflammation caused by the intake of UPF. The inflammation score (INFLA-score), constructed by summarizing the synergistic effects of CRP levels, white blood cell (WBC) count, platelet (PLT) count, and the granulocyte-to-lymphocyte ratio, ranges from −16 to 16 and is used to assess the low-grade inflammation status [22]. Additionally, BMI was included as a mediator in the analysis, considering the causal relationship between diet and BMI. We conducted stratified analyses based on sex, age (<60 and ≥60 years), BMI (<25 and ≥25 kg/m^2^), smoking status (never, former, and current), drinking status (never, former, and current), physical activity (<600 and ≥600 MET-minute/week), and TDI (<median and ≥median). These analyses aimed to further identify individuals who are susceptible to psoriasis. We also performed interaction analyses between UPF intake and the stratified covariates using the likelihood test.

Genotype data from the UK Biobank were generated using a custom Axiom genotyping array that assayed 825,927 genetic variants, followed by genome-wide imputation. The Standard Polygenic Risk Score (PRS) for psoriasis was obtained from the UK Biobank [23], calculated by using a Bayesian approach based on the meta-analyzed summary statistics GWAS data (Field ID: 26269). In our study, the PRS was categorized into low (lowest quintile), intermediate (quintiles 2 to 4), and high (highest quintile) risk.

Sensitivity analyses were conducted by adjusting for cancer, hypertension, diabetes, Crohn’s disease, and cardiovascular disease. We excluded participants who experienced psoriasis events within the first 2 years of follow-up. Additionally, we assessed the effects of replacing the absolute amount of UPF with an equivalent weight of unprocessed or minimally processed food in relation to incident psoriasis. All statistical analyses were carried out using SAS (version 9.4) or R (version 4.2.2).

## 3. Results

In the study, 121,019 participants were included. Of these, 52,731 (43.5%) were males, and 68,288 (56.5%) were females. The average age of the participants was 56.2 years. Over a 12-year follow-up period (with a median follow-up time), 1043 cases of psoriasis were observed.

Table 1 presents the baseline characteristics of all participants from the UK Biobank, categorized by quartiles of the proportion of UPF in their diet. Those with higher UPF consumption tended to be younger, male, and White, as well as former or current smokers and never drinkers. Additionally, individuals with higher levels of UPF intake typically exhibited higher total energy intake, a higher BMI, and lower levels of physical activity.

**Figure 1:**
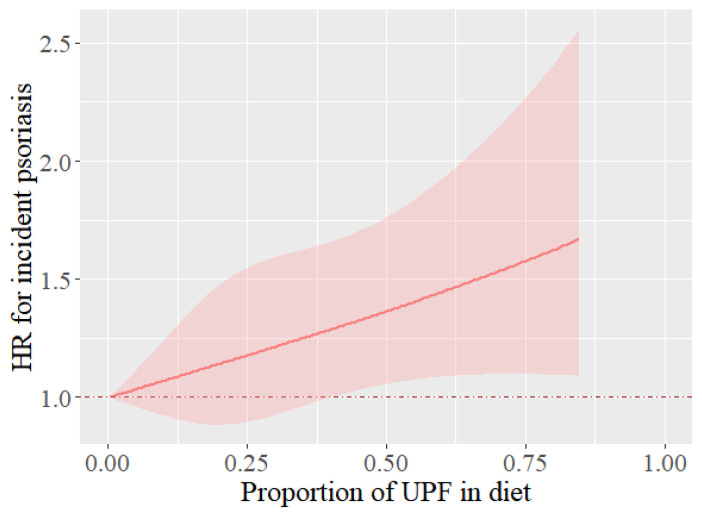
Dose–response association between UPF consumption and risk of incident psoriasis. Adjusted for age, sex, ethnicity, smoking status, drinking status, total energy, physical activity, BMI, and TDI. Abbreviations: HR, hazard ratio.

Table 2 demonstrates a significant positive association between UPF consumption and the risk of psoriasis in different adjusted models. In the fully adjusted model, for every 10% increment in UPF intake within the total diet, HR (95% CI) for psoriasis risk was 1.06 (95% CI: 1.01, 1.11). Additionally, when compared to participants with the lowest UPF consumption, those in the fourth quartile of UPF consumers exhibited a higher risk of psoriasis (HR: 1.28, 95% CI: 1.07–1.52; *p* = 0.006). The restricted cubic spline curve demonstrated an approximately linear association between UPF intake and psoriasis risk (with a *p*-value for non-linear association of 0.90) (Figure 1).

**Figure 2:**
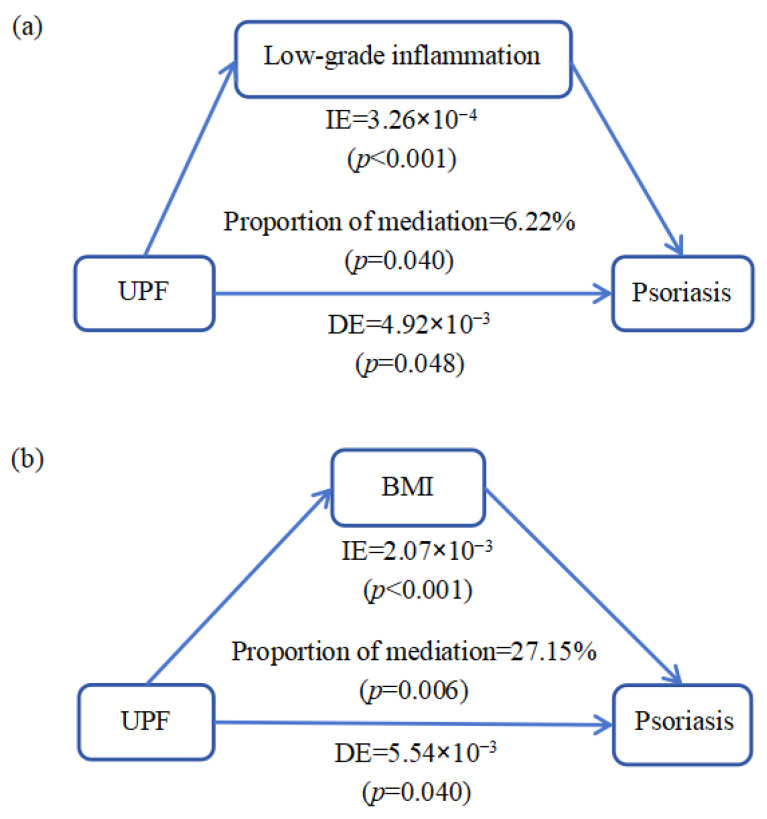
The mediation effect of low-grade inflammation and BMI in the association between UPF consumption and psoriasis. (**a**) INFLA-score mediates the relationship between UPF consumption and psoriasis. Adjusted for age, sex, ethnicity, smoking status, drinking status, total energy, physical activity, BMI, TDI, cancer, hypertension, diabetes, Crohn’s disease, and cardiovascular disease. (**b**) BMI mediates the relationship between UPF consumption and psoriasis. Adjusted for age, sex, ethnicity, smoking status, drinking status, total energy, physical activity, TDI, cancer, hypertension, diabetes, Crohn’s disease, and cardiovascular disease.

The mediation analysis revealed that chronic low-grade inflammation mediated the association between UPF consumption and psoriasis risk (Figure 2). In the entire sample, the total effect of UPF on psoriasis was 5.24 × 10^−3^ (*p* = 0.040), and the indirect effect through INFLA-score was 3.26 × 10^−4^ (*p* < 0.001), explaining 6.22% (*p* = 0.040) of the association between UPF consumption and psoriasis risk (Figure 2). However, when BMI served as a mediator, it explained 27.15% (*p* = 0.006) of the association between UPF consumption and psoriasis risk (Figure 2).

Stratified analysis was performed to further assess the relationship of UPF consumption with the risk of psoriasis among participants with different sex, age, BMI, smoking status, drinking status, level of physical activity, TDI (Table 3), and PRS (Table S6). The results were largely consistent (all *p* interaction > 0.05), while none of the above variables significantly modified the association of UPF intake and psoriasis.

Moreover, our results showed that the risk of psoriasis increased from low to high genetic risk categories (Table S5). In Table 4, we estimated the joint effects of genetic risk and UPF consumption. Among participants with high genetic risk and the highest UPF consumption, the HR for incident psoriasis was 2.73 (95% CI, 1.87–3.98), compared to those with low genetic risk and the lowest UPF consumption. Furthermore, no significant interaction was found between genetic risk and UPF consumption (Table S7).

Sensitivity analysis revealed consistent results (Tables S2–S4). Additionally, in the substitution analysis, replacing 5%, 10%, and 20% of UPF weight in diet with an equivalent proportion of unprocessed or minimally processed foods was associated with a 14% reduction (HR: 0.86; 95% CI: 0.76–0.98; *p* = 0.021), a 17% reduction (HR: 0.83; 95% CI: 0.73–0.95; *p* = 0.005), and an 18% reduction (HR: 0.82; 95% CI: 0.72–0.94; *p* = 0.004) in psoriasis risk, respectively (Table S4).

## 4. Discussion

This study employed a large cohort from the UK Biobank to analyze the association between UPF consumption and psoriasis. After a 12-year follow-up, the results disclosed that a 10% increase in UPF consumption raised the risk of incident psoriasis by 6%. Replacing 20% of UPF weight in the total diet with unprocessed or minimally processed foods decreased the risk of psoriasis by 18% (HR: 0.82; 95% CI: 0.72–0.94; *p* = 0.004). Considering the current high levels and growing consumption of UPF globally, our findings offer robust epidemiological support for its detrimental impact on psoriasis.

To our knowledge, our study constitutes the first large-scale observational investigation into the association between UPF consumption and the incidence of psoriasis, based on data from the UK Biobank. Several studies have already highlighted that UPF contributes to an increased risk of CVD [16], T2D [17], and obesity [24], all of which are common comorbidities related to psoriasis. However, no previously published study has specifically appraised the incidence of psoriasis in connection with UPF consumption, and only a few articles have investigated the association between UPF intake and skin diseases. Given that the notion of UPF has recently garnered substantial attention, we anticipate that more comparable research will surface in the future. In reality, a succession of animal experiments has demonstrated that a Western diet, which contains high amounts of fat and sugar, can bring about psoriasis-like skin inflammation in rodents [25,26]. These findings are in line with our own results. In our dose–response analysis, the restricted cubic spline (RCS) curve indicated that individuals might encounter an increased risk of psoriasis with higher cumulative UPF consumption.

### 4.1. UPF Intake and Elevated BMI

Various potential mechanisms may explain the positive association between UPF consumption and psoriasis. Previous studies have already demonstrated that obesity is probably one of the triggers of psoriasis [27,28]. A meta-analysis has shown that obesity is associated with higher incidence and prevalence of psoriasis, as well as the severity of psoriasis [27]. Several studies have demonstrated that higher consumption of UPFs is significantly associated with increased body weight. Evidence from the French prospective population-based NutriNet-Santé cohort study revealed a positive correlation between UPF intake and elevated BMI [24]. Furthermore, a randomized controlled trial (RCT) conducted in an inpatient setting provided direct evidence that UPFs promote excess calorie intake and subsequent weight gain [29], further supporting this association. Our mediation analysis indicated that the indirect effect of UPF consumption mediated by BMI on psoriasis accounted for 27.15% (*p* = 0.006) of the total effect. Hence, for ordinary people, by restricting the intake of UPF, apart from avoiding the occurrence of obesity, a certain risk of psoriasis can also be reduced.

### 4.2. UPF-Associated Chronic Low-Grade Inflammation

We suspected that another potential mechanism elevating the risk of psoriasis was possibly related to the chronic low-grade inflammation triggered by UPF intake. UPF, typically with high levels of refined sugars and saturated fatty acids, low amounts of dietary fiber or micro-nutrients, and numerous food additives and harmful substances generated during the industrial process, is probably inducing chronic low-grade inflammation within the body [11,30]. Since the energy density of UPF is usually higher than that of unprocessed foods, increasing the consumption of UPF may correspondingly reduce the intake of some anti-inflammatory foods, such as fresh vegetables and fruits, thus further exacerbating the inflammation level. Current epidemiological studies have revealed a potential association between UPF consumption and allergic symptoms (e.g., asthma and eczema) in children [12]. Furthermore, several studies have already indicated that either a diet containing low fiber and high fats or the addition of food additives such as emulsifiers and thickeners can lead to a decreased gut microbial diversity [31,32,33]. However, with the advancements in human micro-biome research, an increasing amount of scientific evidence has emphasized that micro-biome dysbiosis, characterized by altered diversity and composition, is likely to have a negative impact on the regulation of inflammation and immune responses, thereby inducing psoriasis [33]. An animal experiment showed that after ten weeks of continuous Western diet (WD) feeding to mice, in comparison with mice fed the chow diet (CD), the WD-fed mice exhibited a decline in intestinal microbial diversity and significant dysbiosis and were more susceptible to skin inflammation [26]. After the cessation of WD feeding, the inflammation of the mice lessened, and the gut microbiota partially reversed [26]. In our study, the INFLA-score, derived as a sum of four components (including CRP levels, WBC, PLT count, and granulocyte-to-lymphocyte ratio), was employed to evaluate the level of low-grade inflammation [22], and the mediation effect of low-grade inflammation in the association between UPF consumption and psoriasis was 6.22% (*p* = 0.040).

### 4.3. Strengths and Limitations

Our study was a large-scale prospective research study with a long-term follow-up period. We utilized a 24 h dietary recall questionnaire, which provides greater accuracy compared to the food frequency scale. Additionally, we adjusted for multiple confounding factors and conducted a series of sensitivity analyses to ensure the robustness of our findings. However, there are several limitations that need to be acknowledged. Primarily, cases of psoriasis based on patients’ self-reports might lack precision. Nevertheless, given the certain proportion of these self-reports, deleting them may lead to an underestimation of the risk. Secondly, our study population mainly consisted of individuals aged 40 to 69 years, with an average age of 56.2. Notably, this age range corresponds to the second peak of psoriasis onset. Additionally, due to the heterogeneity of psoriatic incidence across different racial groups, our findings may have limited generalizability. Thirdly, while the two 24 h dietary recalls provide valuable snapshots of participants’ dietary intake, this approach has inherent limitations. Most notably, the limited number of recalls may not adequately capture dietary variations or temporal changes occurring throughout the follow-up period. Therefore, we anticipate that more comprehensive data will be available in the future to further validate our findings. Finally, the manual classification of ultra-processed foods is inherently prone to misclassification, which may consequently lead to the underestimation of their adverse health effects.

## 5. Conclusions

In summary, this study was the first to investigate and highlight an increased risk of psoriasis associated with UPF intake. Our research findings suggested that the consumption of UPF was related to an elevated risk of psoriasis, and this connection might be mediated by inflammatory responses and obesity. This underscores the crucial role of controlling UPF intake in the primary prevention of psoriasis.

Compelling epidemiological evidence has established significant associations between ultra-processed food consumption and multiple adverse health outcomes. Based on these findings, our study strongly recommends (1) the inclusion of UPF surveillance metrics in national chronic disease prevention frameworks and (2) the implementation of standardized UPF intake assessment in dermatological practice guidelines for psoriasis management. These evidence-based measures would significantly advance both population-level preventive strategies and individualized patient care. To translate these recommendations into actionable clinical and public health interventions, future research must prioritize the following: (a) establishing causal relationships between UPF exposure and psoriasis incidence through longitudinal study designs; (b) elucidating the pathophysiological mechanisms linking specific UPF components to disease pathogenesis; and (c) developing validated biomarkers for monitoring UPF-related metabolic and inflammatory responses.

## Figures and Tables

**Table 1:** Baseline characteristics by quartile of UPF consumption among UK Biobank participants.

		Quartile of UPF Consumption				*p* Value
	Overall	Q1	Q2	Q3	Q4	
	121,019	30,434	30,366	30,356	29,863	
Proportion of UPF in total diet, % (g/day)						<0.001
Mean (SD)	21.0 (12.3)	8.0 (2.5)	15.0 (1.9)	22.8 (2.6)	38.4 (9.3)	
Age, year						<0.001
Mean (SD)	56.2 (7.8)	56.3 (7.6)	56.4 (7.7)	56.4 (7.9)	55.5 (8.0)	
Sex, *n* (%)						<0.001
Male	52,731 (43.5)	11,739 (38.6)	12,964 (42.7)	13,775 (45.4)	14,253 (47.7)	
Female	68,288 (56.5)	18,695 (61.4)	17,402 (57.3)	16,581 (54.6)	15,610 (52.3)	
Ethnicity, *n* (%)						<0.001
White	116,865 (96.6)	29,073 (95.5)	29,249 (96.3)	29,451 (97.0)	29,092 (97.4)	
Others	4154 (3.4)	1343 (4.5)	1110 (3.7)	900 (3.0)	765 (2.6)	
BMI, kg/m^2^						<0.001
Mean (SD)	26.7 (4.6)	25.9 (4.2)	26.2 (4.3)	26.8 (4.5)	27.8 (5.0)	
Total energy, kj/d						<0.001
Mean (SD)	8703.4 (2032.6)	8224.5 (1955.6)	8788.1 (1987.6)	8952.0 (2018.0)	8852.7 (2088.6)	
Smoking status, *n* (%)						<0.001
Never	69,484 (57.4)	17,044 (56.0)	17,882 (58.9)	17,872 (58.9)	16,686 (55.9)	
Former	43,044 (35.6)	11,478 (37.7)	10,687 (35.2)	10,489 (34.5)	10,390 (34.8)	
Current	8232 (6.8)	1837 (6.0)	1740 (5.7)	1941 (6.4)	2714 (9.1)	
Drinking status, *n* (%)						<0.001
Never	3466 (2.9)	740 (2.4)	820 (2.7)	898 (3.0)	1008 (3.4)	
Former	3413 (2.8)	743(2.4)	814 (2.7)	787 (2.6)	1069 (3.5)	
Current	114,057 (94.2)	28,920 (95.1)	28,712 (94.6)	28,656 (94.3)	27,769 (93.0)	
Physical activity, MET-min/wk						<0.001
Median (IQR)	1678 (933, 2844)	1824 (1040, 2991)	1713 (970, 2862)	1662 (933, 2817)	1506 (813, 2679)	
TDI						<0.001
Median (IQR)	−2.4 (−3.8, −0.1)	−2.0 (−3.6, 0.5)	−2.4 (−3.8, −0.2)	−2.6 (−3.9, −0.4)	−2.4 (−3.8, −0.2)	
INFLA-score						<0.001
Mean (SD)	4.5 (6.1)	4.1 (6.1)	4.4 (6.1)	4.6 (6.0)	5.0 (6.2)	

UPF = ultra-processed food; BMI = body mass index; TDI = Townsend Deprivation Index. Analysis of variance for continuous variables, χ^2^ test for categorical variables.

**Table 2:** Association between UPF consumption and psoriasis, estimated by multivariable Cox proportional hazards regression.

	Continuous ^a^	Quartile of UPF Consumption				*p* for Trend ^b^
		Q1	Q2	Q3	Q4	
Number of cases/non-cases	1043/119,976	277/30,207	241/30,125	276/30,080	299/29,564	
Model 1	1.09 (1.04, 1.14)	Ref.	1.06 (0.88, 1.27)	1.20 (1.01, 1.43)	1.34 (1.13, 1.60)	<0.001
*p*-value	<0.001		0.565	0.042	<0.001	
Model 2	1.08 (1.03, 1.13)	Ref.	1.07 (0.89, 1.28)	1.21 (1.01, 1.44)	1.34 (1.13, 1.60)	<0.001
*p*-value	0.001		0.463	0.035	<0.001	
Model 3	1.06 (1.01, 1.11)	Ref.	1.08 (0.90, 1.29)	1.20 (1.00, 1.44)	1.28 (1.07, 1.52)	0.004
*p*-value	0.015		0.419	0.045	0.006	

Model 1 included age, sex, ethnicity, and the exposure variable (UPF). Model 2 = Model 1 + smoking status (never/ever) and drinking status (never/ever). Model 3 = Model 2 + total energy, body mass index categorized at baseline, physical activity (MET score), and Townsend Deprivation Index. ^a^ Hazard ratio for per increase of 10% in the proportion of UPF intake in the diet. ^b^ Obtained by assigning the median value within each quartile group as a continuous variable.

**Table 3:** Associations between UPF consumption and risk of psoriasis, stratified by main baseline characteristics of participants.

Subgroup	Number of Participants	Hazard Ratio (95%CI)	*p* Value	*p* Interaction
Sex				0.374
Female	68,267	1.07 (1.01, 1.14)	0.048	
Male	52,716	1.02 (0.96, 1.10)	0.506	
Age, years				0.051
<60	71,693	1.08 (1.02, 1.15)	0.012	
≥60	49,290	0.98 (0.91, 1.06)	0.672	
BMI, kg/m^2^				0.068
<25	48,122	0.98 (0.89, 1.08)	0.675	
≥25	72,861	1.09 (1.03, 1.15)	0.002	
Smoking status				0.768
Never	69,484	1.07 (0.99, 1.15)	0.091	
Former	43,044	1.03 (0.96, 1.11)	0.427	
Current	8231	1.04 (0.93, 1.17)	0.523	
Drinking status				0.727
Never	3466	0.94 (0.72, 1.22)	0.623	
Former	3413	1.06 (0.88, 1.27)	0.531	
Current	114,057	1.05 (1.00, 1.10)	0.063	
Physical Activity				0.350
<600	19,047	1.06 (0.95, 1.17)	0.300	
≥600	101,936	1.04 (0.99, 1.10)	0.136	
TDI				0.163
<−2.37	60,544	1.06 (0.98, 1.14)	0.137	
≥−2.37	60,439	1.04 (0.97, 1.10)	0.279	

BMI = body mass index; TDI = Townsend Deprivation Index; UPF = ultra-processed food. Hazard ratio for per increase of 10% in the proportion of UPF consumption in the diet. Adjusted for age, sex, ethnicity, smoking status, drinking status, total energy, physical activity, BMI, and TDI.

**Table 4:** Joint effects of UPF consumption with PRS on the risk of psoriasis.

Subgroup	Cases/Total	Adjusted HRs (95% CI)	*p*-Value
Low genetic risk			
UPF-Q1	37/7291	1 (Reference)	
UPF-Q2	27/7284	0.74 (0.45, 1.22)	0.236
UPF-Q3	46/7385	1.19 (0.77, 1.84)	0.430
UPF-Q4	58/7465	1.45 (0.96, 2.2)	0.076
Medium genetic risk			
UPF-Q1	110/14,736	1.46 (1.01, 2.12)	0.046
UPF-Q2	119/14,859	1.59 (1.10, 2.30)	0.014
UPF-Q3	126/14,812	1.65 (1.14, 2.39)	0.008
UPF-Q4	133/14,293	1.72 (1.19, 2.47)	0.004
High genetic risk			
UPF-Q1	76/7520	2.03 (1.37, 3.01)	<0.001
UPF-Q2	86/7296	2.34 (1.59, 3.45)	<0.001
UPF-Q3	98/7245	2.59 (1.77, 3.80)	<0.001
UPF-Q4	105/7168	2.73 (1.87, 3.98)	<0.001

Models were adjusted for age, sex, ethnicity, total energy intake, body mass index, smoking status, alcohol intake, Townsend Deprivation Index, physical activity, genotyping batch, and genetic principal components. Abbreviations: CI, confidence interval; HR, hazard ratio; PRS, polygenic risk score.

## Data Availability

The UK Biobank resource can be accessed by researchers on application. Data are available from the UK Biobank (https://www.ukbiobank.ac.uk/, accessed on 1 August 2023).

## Supplementary Materials

The following supporting information can be downloaded at: https://www.mdpi.com/article/10.3390/nu17091473/s1. Table S1. UPF items in the Oxford WebQ questionnaire. Table S2. Associations between UPF consumption and risk of psoriasis after excluding psoriasis participants diagnosed within 2 years after baseline. Table S3. Associations between UPF consumption and risk of psoriasis after further adjusting for cancer, hypertension, diabetes, crohn’s disease and cardiovascular disease. Table S4. Association of substituting UPF (%) with unprocessed or minimally processed foods in relation to incident psoriasis. Table S5. Adjusted HRs (95%CIs) for the risk of incident psoriasis by PRS. Table S6. Hazard ratio of psoriasis risk based on UPF consumption stratified by PRS. Table S7. Additive and multiplicative interactions of the UPF consumption with PRS on the risk of incident psoriasis. Figure S1. Selection of participants in the UK Biobank.

## Abbreviations

BMI	body mass index
CI	confidence interval
HR	hazard ratio
INFLA-score	inflammation score
PRS	polygenic risk score
TDI	Townsend Deprivation Index
UPF	ultra-processed food

## References

[1] Armstrong A.W., Read C. (2020). Pathophysiology, clinical presentation, and treatment of psoriasis: A review. JAMA.

[2] Griffiths C.E.M., Armstrong A.W., Gudjonsson J.E., Barker J. (2021). Psoriasis. Lancet.

[3] Parisi R., Iskandar I.Y.K., Kontopantelis E., Augustin M., Griffiths C.E.M., Ashcroft D.M. (2020). National, regional, and worldwide epidemiology of psoriasis: Systematic analysis and modelling study. BMJ.

[4] Damiani G., Bragazzi N.L., Karimkhani Aksut C., Wu D., Alicandro G., McGonagle D., Guo C., Dellavalle R., Grada A., Wong P. (2021). The global, regional, and national burden of psoriasis: Results and insights from the global burden of disease 2019 study. Front. Med..

[5] Armstrong E.J., Harskamp C.T., Armstrong A.W. (2013). Psoriasis and major adverse cardiovascular events: A systematic review and meta-analysis of observational studies. J. Am. Heart Assoc..

[6] Eppinga H., Poortinga S., Thio H.B., Nijsten T.E.C., Nuij V., van der Woude C.J., Vodegel R.M., Fuhler G.M., Peppelenbosch M.P. (2017). Prevalence and phenotype of concurrent psoriasis and inflammatory bowel disease. Inflamm. Bowel Dis..

[7] Singh S., Taylor C., Kornmehl H., Armstrong A.W. (2017). Psoriasis and suicidality: A systematic review and meta-analysis. J. Am. Acad. Dermatol..

[8] Dowlatshahi E.A., Wakkee M., Arends L.R., Nijsten T. (2014). The prevalence and odds of depressive symptoms and clinical depression in psoriasis patients: A systematic review and meta-analysis. J. Investig. Dermatol..

[9] Dalgard F.J., Gieler U., Tomas-Aragones L., Lien L., Poot F., Jemec G.B.E., Misery L., Szabo C., Linder D., Sampogna F. (2015). The psychological burden of skin diseases: A cross-sectional multicenter study among dermatological out-patients in 13 european countries. J. Investig. Dermatol..

[10] Katsimbri P., Korakas E., Kountouri A., Ikonomidis I., Tsougos E., Vlachos D., Papadavid E., Raptis A., Lambadiari V. (2021). The effect of antioxidant and anti-inflammatory capacity of diet on psoriasis and psoriatic arthritis phenotype: Nutrition as therapeutic tool?. Antioxidants.

[11] Tristan Asensi M., Napoletano A., Sofi F., Dinu M. (2023). Low-grade inflammation and ultra-processed foods consumption: A review. Nutrients.

[12] Kong W., Xie Y., Zhong J., Cao C. (2022). Ultra-processed foods and allergic symptoms among children and adults in the united states: A population-based analysis of nhanes 2005–2006. Front. Public Health.

[13] Wang L., Martínez Steele E., Du M., Pomeranz J.L., O’Connor L.E., Herrick K.A., Luo H., Zhang X., Mozaffarian D., Zhang F.F. (2021). Trends in consumption of ultraprocessed foods among us youths aged 2–19 years, 1999–2018. JAMA.

[14] Popkin B.M., Barquera S., Corvalan C., Hofman K.J., Monteiro C., Ng S.W., Swart E.C., Taillie L.S. (2021). Towards unified and impactful policies to reduce ultra-processed food consumption and promote healthier eating. Lancet Diabetes Endocrinol..

[15] Baker P., Machado P., Santos T., Sievert K., Backholer K., Hadjikakou M., Russell C., Huse O., Bell C., Scrinis G. (2020). Ultra-processed foods and the nutrition transition: Global, regional and national trends, food systems transformations and political economy drivers. Obes. Rev..

[16] Chen X., Chu J., Hu W., Sun N., He Q., Liu S., Feng Z., Li T., Han Q., Shen Y. (2022). Associations of ultra-processed food consumption with cardiovascular disease and all-cause mortality: Uk biobank. Eur. J. Public Health.

[17] Levy R.B., Rauber F., Chang K., Louzada M., Monteiro C.A., Millett C., Vamos E.P. (2021). Ultra-processed food consumption and type 2 diabetes incidence: A prospective cohort study. Clin. Nutr..

[18] Lo C.H., Khandpur N., Rossato S.L., Lochhead P., Lopes E.W., Burke K.E., Richter J.M., Song M., Ardisson Korat A.V., Sun Q. (2022). Ultra-processed foods and risk of crohn’s disease and ulcerative colitis: A prospective cohort study. Clin. Gastroenterol. Hepatol..

[19] Hawkins P., Earl K., Tektonidis T.G., Fallaize R. (2024). The role of diet in the management of psoriasis: A scoping review. Nutr. Res. Rev..

[20] Greenwood D.C., Hardie L.J., Frost G.S., Alwan N.A., Bradbury K.E., Carter M., Elliott P., Evans C.E.L., Ford H.E., Hancock N. (2019). Validation of the oxford webq online 24-h dietary questionnaire using biomarkers. Am. J. Epidemiol..

[21] Monteiro C.A., Cannon G., Levy R.B., Moubarac J.C., Louzada M.L., Rauber F., Khandpur N., Cediel G., Neri D., Martinez-Steele E. (2019). Ultra-processed foods: What they are and how to identify them. Public Health Nutr..

[22] Pounis G., Bonaccio M., Di Castelnuovo A., Costanzo S., de Curtis A., Persichillo M., Sieri S., Donati M.B., Cerletti C., de Gaetano G. (2016). Polyphenol intake is associated with low-grade inflammation, using a novel data analysis from the moli-sani study. Thromb. Haemost..

[23] Thompson D.J., Wells D., Selzam S., Peneva I., Moore R., Sharp K., Tarran W.A., Beard E.J., Riveros-Mckay F., Giner-Delgado C. (2022). Uk biobank release and systematic evaluation of optimised polygenic risk scores for 53 diseases and quantitative traits. medRxiv.

[24] Beslay M., Srour B., Mejean C., Alles B., Fiolet T., Debras C., Chazelas E., Deschasaux M., Wendeu-Foyet M.G., Hercberg S. (2020). Ultra-processed food intake in association with bmi change and risk of overweight and obesity: A prospective analysis of the french nutrinet-sante cohort. PLoS Med..

[25] Shi Z., Wu X., Yu S., Huynh M., Jena P.K., Nguyen M., Wan Y.Y., Hwang S.T. (2020). Short-term exposure to a western diet induces psoriasiform dermatitis by promoting accumulation of il-17a-producing gammadelta t cells. J. Investig. Dermatol..

[26] Shi Z., Wu X., Santos Rocha C., Rolston M., Garcia-Melchor E., Huynh M., Nguyen M., Law T., Haas K.N., Yamada D. (2021). Short-term western diet intake promotes il-23–mediated skin and joint inflammation accompanied by changes to the gut microbiota in mice. J. Investig. Dermatol..

[27] Barros G., Duran P., Vera I., Bermúdez V. (2022). Exploring the links between obesity and psoriasis: A comprehensive review. Int. J. Mol. Sci..

[28] Paroutoglou K., Papadavid E., Christodoulatos G.S., Dalamaga M. (2020). Deciphering the association between psoriasis and obesity: Current evidence and treatment considerations. Curr. Obes. Rep..

[29] Hall K.D., Ayuketah A., Brychta R., Cai H., Cassimatis T., Chen K.Y., Chung S.T., Costa E., Courville A., Darcey V. (2019). Ultra-processed diets cause excess calorie intake and weight gain: An inpatient randomized controlled trial of ad libitum food intake. Cell. Metab..

[30] Ma X., Nan F., Liang H., Shu P., Fan X., Song X., Hou Y., Zhang D. (2022). Excessive intake of sugar: An accomplice of inflammation. Front. Immunol..

[31] Chassaing B., Compher C., Bonhomme B., Liu Q., Tian Y., Walters W., Nessel L., Delaroque C., Hao F., Gershuni V. (2022). Randomized controlled-feeding study of dietary emulsifier carboxymethylcellulose reveals detrimental impacts on the gut microbiota and metabolome. Gastroenterology.

[32] Bancil A.S., Sandall A.M., Rossi M., Chassaing B., Lindsay J.O., Whelan K. (2021). Food additive emulsifiers and their impact on gut microbiome, permeability, and inflammation: Mechanistic insights in inflammatory bowel disease. J. Crohns. Colitis..

[33] Kapoor B., Gulati M., Rani P., Gupta R. (2022). Psoriasis: Interplay between dysbiosis and host immune system. Autoimmun. Rev..

