# Supplementary material for: Ultra-Processed Food Consumption and the Risk of Psoriasis: A Large Prospective Cohort Study
Source: Nutrients. 2025 Apr 27;17(9):1473. doi: 10.3390/nu17091473 (PMC12073620; doi:10.3390/nu17091473)

Supplementary materials

Table S1. UPF items in the Oxford WebQ questionnaire.
Table S2. Associations between UPF consumption and risk of psoriasis after excluding. psoriasis participants diagnosed within 2 years after baseline.
Table S3. Associations between UPF consumption and risk of psoriasis after further adjusting for cancer, hypertension, diabetes, crohn's disease and cardiovascular disease.
Table S4. Association of substituting UPF (%) with unprocessed or minimally processed foods in relation to incident psoriasis.
Table S5. Adjusted HRs (95%CIs) for the risk of incident psoriasis by PRS.
Table S6. Hazard ratio of psoriasis risk based on UPF consumption stratified by PRS.
Table S7. Additive and multiplicative interactions of the UPF consumption with PRS on the risk of incident psoriasis.
Figure S1. Selection of participants in the UK Biobank.

Table S1. UPF items in the Oxford WebQ questionnaire.

**UPF items**
Low calorie drink, Fizzy drink intake carbonated (fizzy) drinks, Squash, Instant coffee, Artificial sweetener added to cereal, Artificial sweetener added to coffee, Artificial sweetener added to tea, Flavoured milk, Fortified wine, Spirits, Porridge, Muesli, Oat crunch, Sweetened cereal, Plain cereal, Bran cereal, Whole-wheat cereal, Other cereal, Bap, Bread roll, Naan bread, Crispbread, Oatcakes, Other bread, Number of bread slices with butter/margarine, Number of baguettes with butter/margarine, Number of baps with butter/margarine, Number of bread rolls with butter/margarine, Number of crackers/crispbreads with butter/margarine, Number of oatcakes with butter/margarine, Number of other bread types with butter/margarine, Double crust pastry, Single crust pastry, Crumble, Pizza, Pancake, Scotch pancake, Yorkshire pudding, Indian snacks, Croissant, Danish pastry, Scone, Ice-cream, Milk-based pudding intake, Other milk-based pudding intake, Soya dessert, Fruitcake, Cake, Doughnut, Sponge pudding, Cheesecake, Other dessert, Chocolate bar, White chocolate, Milk chocolate, Dark chocolate, Chocolate-covered raisin, Chocolate sweet, Diet sweets, Sweets, Chocolate-covered biscuits, Chocolate biscuits, Sweet biscuits, Cereal bar, Other sweets intake sweet snacks, Crisp, Savoury biscuits, Cheesy biscuits, Other savoury snack, Powdered/instant soup, Snackpot, Couscous, Low fat cheese spread, Cheese spread, Sausage, Crumbed or deep-fried poultry, Bacon, Ham, Vegetarian sausages/burgers, Quorn, Other vegetarian alternative, Baked bean, Fried potatoes, Mashed

Table S2. Associations between UPF consumption and risk of psoriasis after excluding psoriasis participants diagnosed within 2 years after baseline.

	Continuous[a]	Quartile of UPF consumption				*P* for trend[b]
		1st quartile	2nd quartile	3rd quartile	4th quartile	
Number of cases/non-cases	886/119936	198/29998	201/29916	223/29847	264/29286	
Model 1	1.10(1.04,1.16)	Ref.	1.01(0.83,1.23)	1.11(0.92,1.35)	1.36(1.13,1.64)	<0.001
P-value	<0.001		0.921	0.276	0.001	
Model 2	1.09(1.04,1.15)	Ref.	1.03(0.84,1.25)	1.12(0.92,1.36)	1.36(1.13,1.64)	<0.001
P-value	<0.001		0.800	0.248	0.001	
Model 3	1.07(1.02,1.13)	Ref.	1.04(0.85,1.27)	1.12(0.92,1.36)	1.30(1.08,1.57)	0.002
P-value	0.007		0.710	0.257	0.006	

Model 1 included age, sex, ethnicity and the exposure variable (UPF).

Model 2= Model 1 + smoking status (never/ever) and drinking status (never/ever).

Model 3= Model 2 + total energy, BMI categorized at baseline, physical activity (MET score) and Townsend Deprivation Index.

[a] Hazard ratio for per increase of 10% in the proportion of UPF intake in the diet.

[b] Obtained by assigning the median value within each quartile group as a continuous variable.

Table S3. Associations between UPF consumption and risk of psoriasis after further adjusting for cancer, hypertension, diabetes, crohn's disease and cardiovascular disease.

		Quartile of UPF consumption				
	Continuous[a]	1st quartile	2nd quartile	3rd quartile	4th quartile	*P* for trend[b]
Number of cases/non-cases	1043/119976	227/30207	241/30125	276/30080	299/29564	
Model	1.67 (1.04, 2.70)	Ref.	1.07 (0.89, 1.29)	1.19 (0.99, 1.42)	1.25 (1.05, 1.49)	0.009
P-value	0.034		0.446	0.057	0.012	

Models were adjusted for age, sex, body mass index, ethnicity, Townsend deprivation index, smoking status, alcohol intake, physical activity, total energy, cancer, hypertension, diabetes, crohn’s disease and cardiovascular disease.

[a] Hazard ratio for per increase of 10% in the proportion of UPF intake in the diet.

[b] Obtained by assigning the median value within each quartile group as a continuous variable.

Table S4. Association of substituting UPF (%) with unprocessed or minimally processed foods in relation to incident psoriasis.

	Model 1		Model 2		Model 3	
	HR (95% CI)	*P*-value	HR (95% CI)	*P*-value	HR (95% CI)	*P*-value
Replacing 5% of UPF weight in total diet with an equivalent proportion of UNP	0.83 (0.73,0.94)	0.004	0.84 (0.74,0.95)	0.006	0.86 (0.76,0.98)	0.021
Replacing 10% of UPF weight in total diet with an equivalent proportion of UNP	0.80 (0.70,0.91)	<0.001	0.81 (0.71,0.92)	0.001	0.83 (0.73,0.95)	0.005
Replacing 20% of UPF weight in total diet with an equivalent proportion of UNP	0.79 (0.64,0.91)	<0.001	0.80 (0.70,0.92)	0.001	0.82 (0.72,0.94)	0.004

Model 1 included age, sex, ethnicity and the exposure variable (UPF).

Model 2= Model 1 + smoking status (never/ever) and drinking status (never/ever).

Model 3= Model 2 + total energy, BMI categorized at baseline, physical activity (MET score) and Townsend Deprivation Index.

Abbreviations: UPF, ultra-processed foods; UNP, unprocessed or minimally processed foods; HR, hazard ratio; CI, confidence interval.

Table S5. Adjusted HRs (95%CIs) of the risk of incident psoriasis by PRS.

	PRS<br>Hazard ratio (95%CI)	*P*-value
Cases/total	1018/121019	
Continuous	1.31 (1.24,1.39)	<0.001
Low genetic risk	1 (Reference)	
Medium genetic risk	1.45 (1.22,1.73)	<0.001
High genetic risk	2.20 (1.83,2.65)	<0.001
P for trend	<0.001	

Models were adjusted for age, sex, ethnicity, total energy intake, body mass index, smoking status, alcohol intake, Townsend deprivation index, physical activity, genotyping batch, and genetic principal components.

Abbreviations: CI, confidence interval; PRS, polygenic risk score.

Table S6. Hazard ratio of psoriasis risk based on UPF consumption stratified by PRS.

	Low genetic risk		Medium genetic risk		High genetic risk	
	Hazard ratio (95% CI)	*P*-value	Hazard ratio (95% CI)	*P*-value	Hazard ratio (95% CI)	*P*-value
Cases/total	132/23669		592/71009		296/23669	
Continuous	1.09 (0.95,1.24)	0.212	1.02 (0.95,1.08)	0.613	1.08 (0.99,1.18)	0.100
Q1	1 (Reference)		1 (Reference)		1 (Reference)	
Q2	0.80 (0.45,1.43)	0.454	1.10 (0.87,1.39)	0.451	1.05 (0.74,1.49)	0.796
Q3	1.45 (0.88,2.40)	0.146	1.10 (0.87,1.39)	0.441	1.25 (0.90,1.76)	0.189
Q4	1.61 (0.98,2.63)	0.060	1.09 (0.87,1.38)	0.453	1.31 (0.94,1.82)	0.113
P for trend	0.012		0.536		0.072	

Models were adjusted for age, sex, ethnicity, total energy intake, body mass index, smoking status, alcohol intake, Townsend deprivation index, physical activity, genotyping batch, and genetic principal components.

Abbreviations: CI, confidence interval; PRS, polygenic risk score.

Table S7. Additive and multiplicative interactions of the UPF consumption with PRS on the risk of incident psoriasis.

	RERI(95%CI)	AP(95%CI)	*P*-value additive interactions	*P*-Value multiplicative interactions
UPF consumption	-0.01 (-0.06,0.06)	-0.01 (-0.03,0.03)	0.996	0.440

Models were adjusted for age, sex, ethnicity, total energy intake, body mass index, smoking status, alcohol intake, Townsend deprivation index, physical activity, genotyping batch, and genetic principal components.

Abbreviations: CI, confidence interval; PRS, polygenic risk score; RERI, relative excess risk due to interaction; AP, attributable proportion due to interaction.

Figure S1. Selection of participants in the UK Biobank.

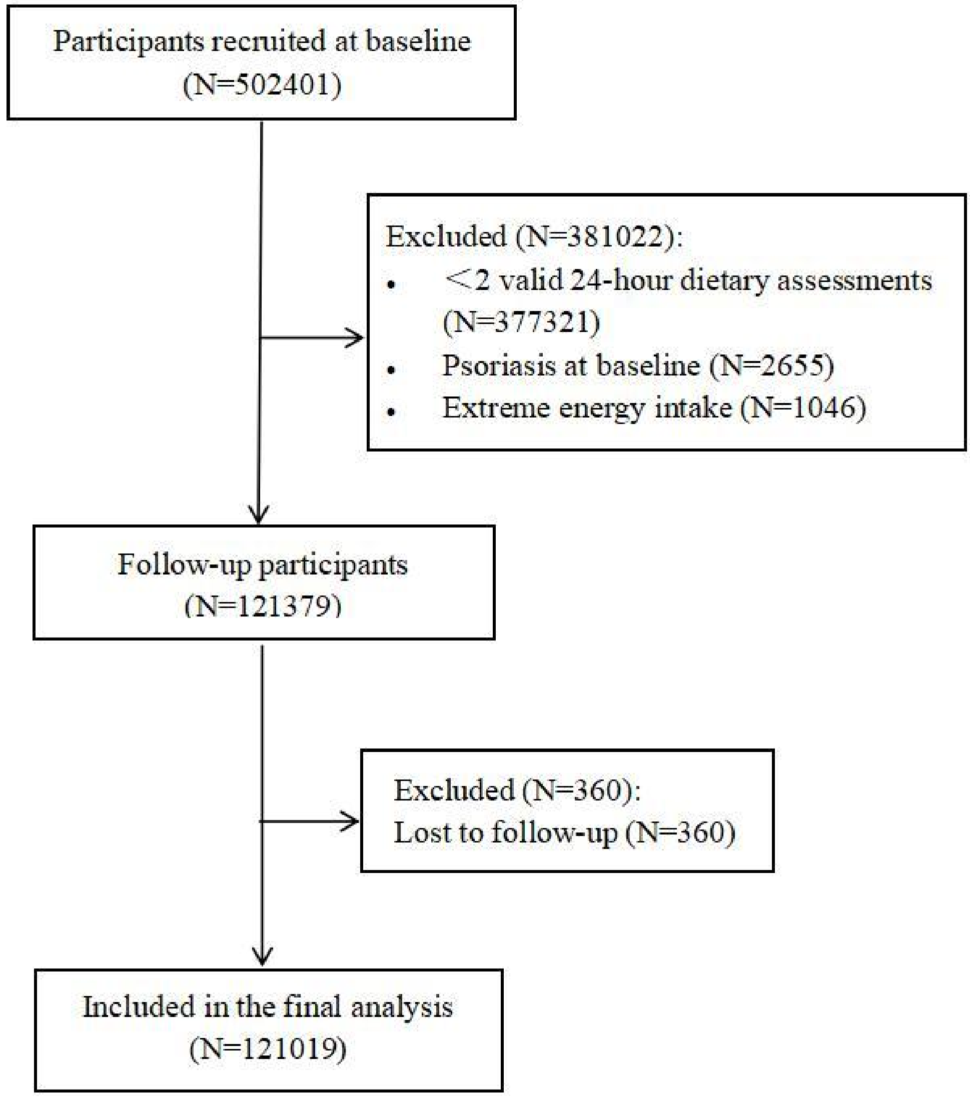

Supplement: Supplementary file 1 [file nutrients-17-01473-s001.zip › nutrients-3579209-supplementary.pdf]
